# Diversity in Functional Organization of Class I and Class II Biotin Protein Ligase

**DOI:** 10.1371/journal.pone.0016850

**Published:** 2011-03-03

**Authors:** Sudha Purushothaman, Karthikeyan Annamalai, Anil K. Tyagi, Avadhesha Surolia

**Affiliations:** 1 Molecular Biophysics Unit, Indian Institute of Science, Bangalore, India; 2 National Institute of Immunology, Aruna Asaf Ali Marg, New Delhi, India; 3 Department of Biochemistry, University of Delhi South Campus, New Delhi, India; Fundació Institut Germans Trias i Pujol, Universitat Autònoma de Barcelona CibeRES, Spain

## Abstract

The cell envelope of *Mycobacterium tuberculosis* (*M.tuberculosis*) is composed of a variety of lipids including mycolic acids, sulpholipids, lipoarabinomannans, etc., which impart rigidity crucial for its survival and pathogenesis. Acyl CoA carboxylase (ACC) provides malonyl-CoA and methylmalonyl-CoA, committed precursors for fatty acid and essential for mycolic acid synthesis respectively. Biotin Protein Ligase (BPL/BirA) activates apo-biotin carboxyl carrier protein (BCCP) by biotinylating it to an active holo-BCCP. A minimal peptide (Schatz), an efficient substrate for *Escherichia coli* BirA, failed to serve as substrate for *M. tuberculosis* Biotin Protein Ligase (*Mt*BPL). *Mt*BPL specifically biotinylates homologous BCCP domain, *Mt*BCCP_87_, but not *Ec*BCCP_87_. This is a unique feature of *Mt*BPL as *Ec*BirA lacks such a stringent substrate specificity. This feature is also reflected in the lack of self/promiscuous biotinylation by *Mt*BPL. The N-terminus/HTH domain of *Ec*BirA has the self-biotinable lysine residue that is inhibited in the presence of Schatz peptide, a peptide designed to act as a universal acceptor for *Ec*BirA. This suggests that when biotin is limiting, *Ec*BirA preferentially catalyzes, biotinylation of BCCP over self-biotinylation. R118G mutant of *Ec*BirA showed enhanced self and promiscuous biotinylation but its homologue, R69A *Mt*BPL did not exhibit these properties. The catalytic domain of *Mt*BPL was characterized further by limited proteolysis. Holo-*Mt*BPL is protected from proteolysis by biotinyl-5′ AMP, an intermediate of *Mt*BPL catalyzed reaction. In contrast, apo-*Mt*BPL is completely digested by trypsin within 20 min of co-incubation. Substrate selectivity and inability to promote self biotinylation are exquisite features of *Mt*BPL and are a consequence of the unique molecular mechanism of an enzyme adapted for the high turnover of fatty acid biosynthesis.

## Introduction

*Mycobacterium tuberculosis* has become resistant to most drugs. The cell wall, composed of almost 60% lipids that are long chain, branched fatty acids, is highly hydrophobic and hence refractory to several components of human defense system. It also provides an effective permeability barrier against several anti-mycobacterial agents [1]–[3]. The rich diversity of lipids present in *M. tuberculosis* is reflected at the genomic level by a large repertoire of genes for lipid biosynthesis. *M. tuberculosis*, for example, has ∼300 enzymes involved in lipid synthesis while *E. coli* has only about 50 [4]–[7].

**Figure 1:**
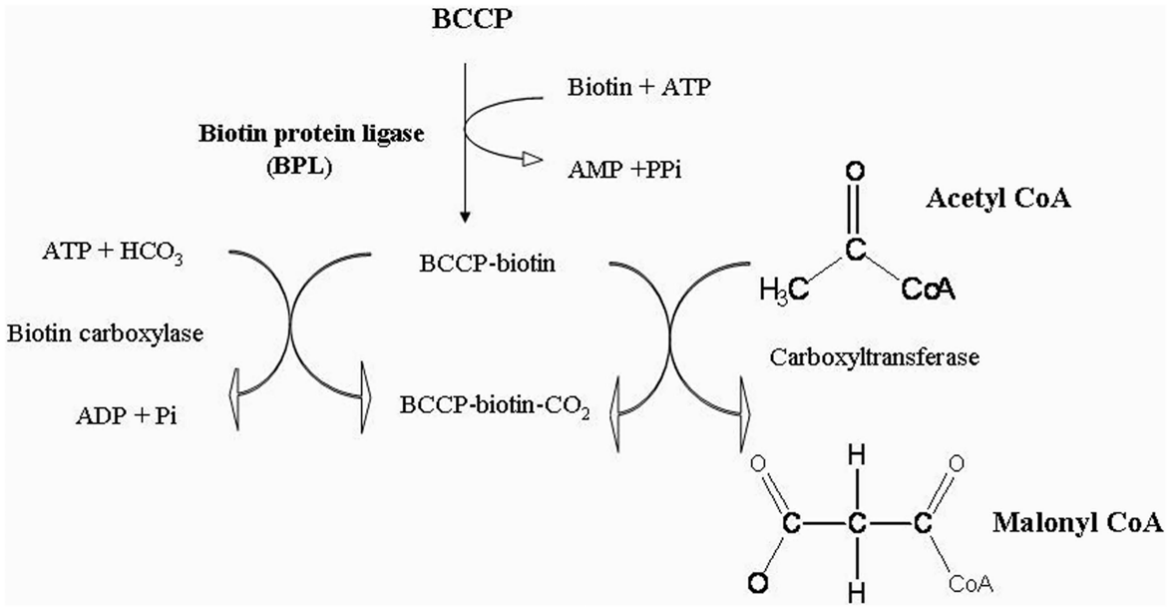
Schematic outline of the functional cycle of the BCCP subunit of acetyl CoA carboxylase. The BCCP is involved in three homologous protein-protein interactions with the Biotin Protein Ligase (BPL), Biotin carboxylase (BC) and Carboxyl transferase (CT).

Biotin-dependent enzymes are involved in carboxylation and decarboxylation reactions. Acyl CoA carboxylases (ACC) catalyze biotin-dependent carboxylation of nascent molecules such as acetyl-CoA, propionyl-CoA etc. These carboxylases are multi-subunit, multi-domain proteins consisting of α and β subunits. *M. tuberculosis* has three copies of α-subunits which are composed of a N-terminus biotin carboxylase (BC) and a C-terminus biotin carboxyl carrier protein (BCCP). All biotinyl domains so far reported have a target lysine at −35^th^ residue from C-terminus for biotinylation [8]. Hence, a protein composed of C-terminus 87 amino acids of *acc* is an efficient substrate for Biotin Protein ligase [8]. The β-subunit has carboxyl transferase (CT) activity [8]. Biotinylation of BCCP is catalyzed by Biotin Protein Ligase (BPL) which promotes an amide linkage between the carboxyl group of biotin and the ε-amino group of a specific lysine residue nestled within a conserved ‘AMKM’ sequence of BCCP. Biotinylation converts inactive apo-BCCP to functional holo-BCCP that participates in the transcarboxylation reaction [9], [10]. Thus, BCCP has two functions - ***mechanistic*** by serving as carboxyl carrier in overall carboxylation reaction and ***structural***, by swinging carboxybiotin to the carboxyl transferase component of ACC. BC carboxylates the ureido nitrogen atom of biotin covalently bound to BCCP which moves {CO_2_}-biotin to the active site of carboxyl transferase (CT), for the transfer of a carboxyl group to acetyl or propionyl CoA [11], [12]. The entire sequence of carboxylation reaction and the key role played by BCCP is schematically represented in Figure 1.

In spite of a highly conserved function, BCCPs display unique features for their respective biotinylating enzymes. In solution, apo-BCCP (*E. coli*, *Pyrococcus horikoshii*) is a flattened β- barrel structure comprising of two four-stranded β sheets [12], [13]. In most BCCPs, the biotinable lysine is nestled within the conserved tetrapeptide ‘AMKM’ sequence in an exposed β-turn of BCCP domain. However, in *Sulfolobus tokodaii*, the canonical lysine residue within the sequence ‘AMKS’ was not biotinylated by *Ec*BirA [14], [15]. In *Aquifex aeolicus* (*Aa*BPL), the target lysine is within the ‘ALKV’ sequence [16]. BCCP of *M. tuberculosis* (*Mt*BCCP) is part of a multi-domain enzyme, biotin carboxylase and this probably alters its dynamics with the cognate enzyme, *Mt*BPL.

*Mt*BPL belongs to class I BPLs which lack a DNA binding domain at their N-termini unlike the class II BPLs (e.g. *Ec*BirA) hence are devoid of repressor function exhibited by class II BPLs [17]–[19]. Our previous study showed that the two enzymes differ in several ways from structural organization to ligand interactions [20]. *Ec*BirA can biotinylate BCCPs of other species. *Mt*BPL as shown in this study, in contrast, to *Ec*BirA exhibits exquisite substrate specificity. The differences in their activities are correlated here with their intrinsic metabolic functions.

## Results

### Protein purification

It has been reported that the C-terminus domain of BCCP (apo-BCCP_87_), does not self-associate and was a good substrate for biotinylation reaction [11], [12]. Hence *Ec*BCCP_87_ and *Mt*BCCP_87_ expressed in pET28a were used for avidin blot assays. The BCCP was purified by Ni-NTA column chromatography. The apo form was separated from the holo form using a Mono Q column pre-equilibrated with 10 mM Tris-HCl buffer (pH- 8.0) prior to the elution of the protein with a salt gradient (0–100% 10 mM Tris-HCl pH-8.0, 1 M NaCl). Fractions containing apo-BCCP were checked on avidin blot, pooled and dialyzed against 10 mM Tris-HCl pH 8.0, 50 mM KCl, 2.5 mM MgCl_2_ (standard buffer). Thus ∼95% of the purified *Mt*BCCP_87_/*Ec*BCCP_87_was found to be in their apo form. The biotinylation reaction was found to be dependent on Mg^2+^, ATP and biotin. BCCP and BPL were dialyzed against the standard buffer prior to use.

For self-biotinylation assays, BL21 containing *Ec*BirA construct was grown in M9 media supplemented with 2% glucose for 5 h and induced for 3 h to prevent endogenous self-biotinylation. The eluted protein was dialyzed, concentrated and dialyzed against standard buffer.

### Domain architecture of Biotin protein ligase

**Figure 2:**
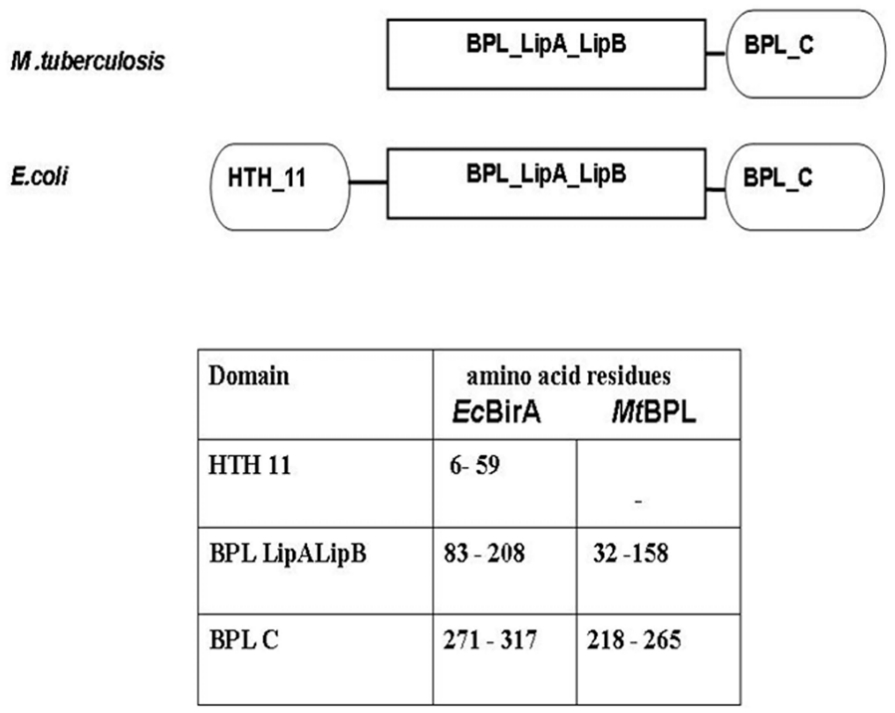
Domain architecture of Class I and II BPLs. The domains were designed from pfam results. *Mt*BPL belongs to Class I and *Ec*BirA belongs to Class II family of BPLs.

The domain structure of *Mt*BPL and *Ec*BirA was obtained from pfam (Figure 2) [21]. The different domains of BPL are:

#### HTH

The helix-turn helix domain.

#### BPL_LipA_ LipB

This family includes biotin protein ligase, lipoate-protein ligase A and B.

#### BPL C

The C-terminus domain has a SH3-like barrel fold, the function of which is unknown. BPL family is a member of clan *TRB* (Transcriptional repressor beta-barrel domain).This beta-barrel domain is found at the C-terminus of a variety of transcriptional repressor proteins. As shown in the Figure 2, Biotin Protein Ligase of *M.tuberculosis* lacks the N-terminus HTH domain and hence does not function as a repressor.

### Substrate specificity of *Mt*BPL

**Figure 3:**
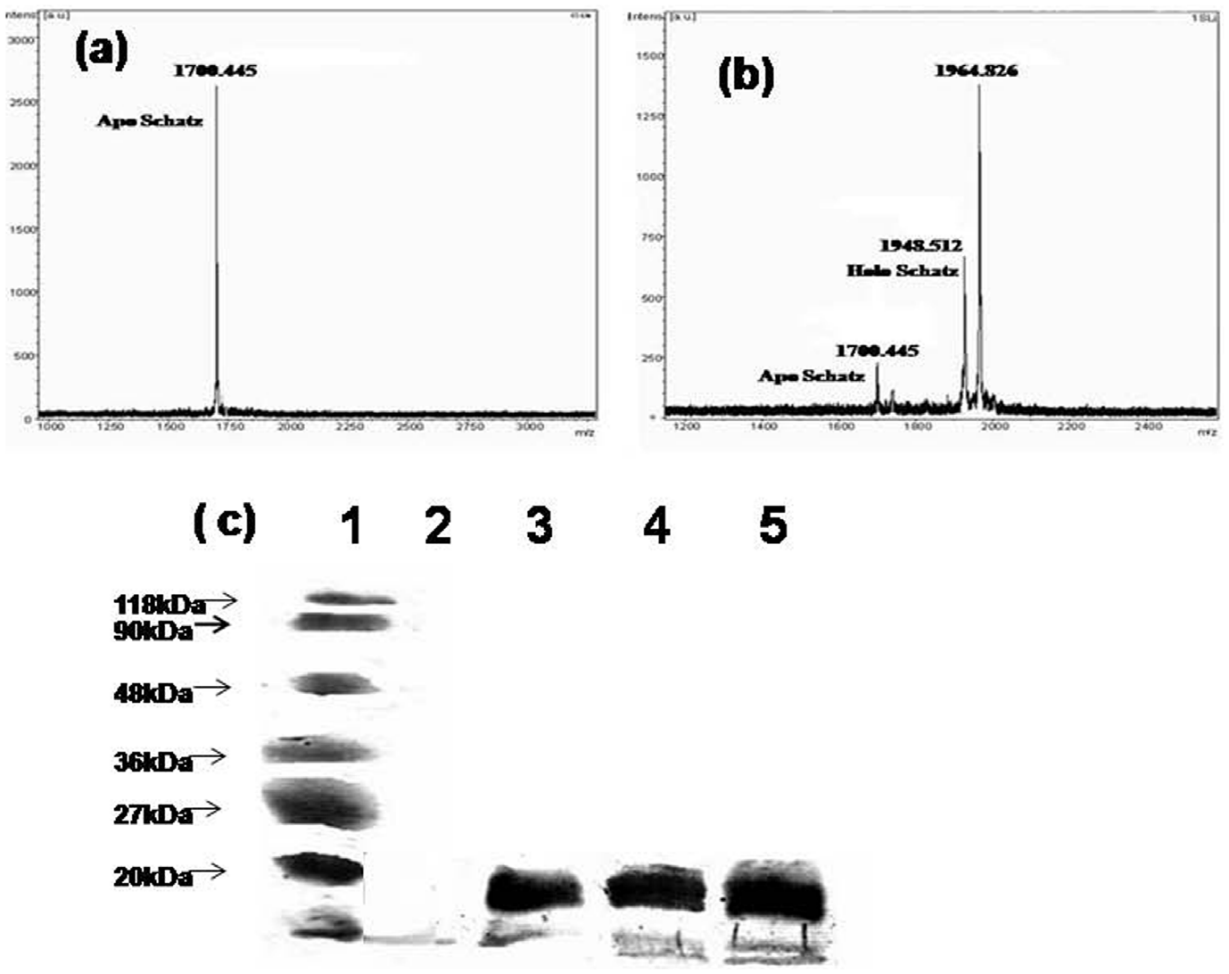
Biotinylation by *Mt*BPL or *Ec*BirA. (a) Mass spectrum of Schatz peptide incubated with 500 µM biotin, 3 mM ATP and 100 nM *Mt*BPL or *Ec*BirA in standard buffer. (b) Mass spectrum of Schatz peptide incubated with 500 µM biotin, 3 mM ATP and 100 nM *Ec*BirA in standard buffer. (c) Avidin blot of biotinylation of BCCP catalyzed by BPL. The reaction was carried in standard buffer (10 mM Tris-HCl pH-8.0, 50 mM KCl, 2.5 mM MgCl_2_) containing 3 mM ATP, 500 µM biotin, 2.5 mM MgCl_2_, 0.1 mM dithiothreitol, and 100 nM BPL and 5 µM BCCP_87_ for 30 min at 37°C. The reaction mixture was then resolved on a 10% SDS PAGE and transferred to nitrocellulose membrane. The membrane was then incubated with streptavidin HRP for 1 h at room temperature and developed with AEC/H_2_O_2 (1) marker; (2) *Ec*BCCP87+*Mt*BPL; (3) *Mt*BCCP87+*Mt*BPL_; (4) *Ec*BCCP_87_+*Ec*BirA; (5) *Mt*BCCP_87_+*Ec*BirA.

The molecular behavior of *Mt*BPL and *Ec*BirA are different due to the presence of an additional repressor function in *Ec*BirA. It has been documented that *Ec*BirA biotinylates BCCPs from other species except the one from *S. tokadii* [15]. In fact, *Ec*BirA efficiently biotinylated the synthetic biotinable minimal peptide of sequence ‘GLNDIFEAQKIEWH’ (Schatz peptide) which is known to be a good substrate for BPLs (Figure 3b). In contrast, *Mt*BPL failed to biotinylate Schatz peptide (Figure 3a). Subsequently, we investigated the ability of *Mt*BPL and *Ec*BirA to cross biotinylate *Ec*BCCP_87_ and *Mt*BCCP_87_. BCCPs (5 µM) were incubated with 500 µM biotin, 3 mM ATP, 100 nM *Ec*BirA or *Mt*BPL for 30 min at 37°C. *Ec*BirA efficiently biotinylated both the BCCPs but *Mt*BPL selectively biotinylated its cognate substrate (*Mt*BCCP_87_) alone and failed to biotinylate *Ec*BCCP_87_ (Figure 3c).

### Self-biotinylation of *Ec*BirA

When substrate specificity of BPLs was explored, at higher enzyme concentration, a protein with molecular weight corresponding to *Ec*BirA was detected on avidin blot indicating that *Ec*BirA undergoes self-biotinylation. This is consistent with the report of Choi-Rhee *et al* [22]. Therefore, we investigated if *Mt*BPL was capable of self-biotinylation like its counterpart in *E. coli. Mt*BPL or *Ec*BirA (250–2000 nM) were subjected to biotinylation reaction for 1 h in the absence of BCCP. The biotinylation mixture was resolved on 12% SDS-PAGE, transferred onto nitrocellulose membrane and detected by streptavidin HRP. The control, *Ec*BirA was self-biotinylated at concentration as low as 500 nM (Fig S1). In contrast, *Mt*BPL did not undergo self-biotinylation even at 2000 nM (Lane 2–6, Fig S1). Hence, our focus was to study the implications of the lack of self- biotinylation in *Mt*BPL.

**Figure 4:**
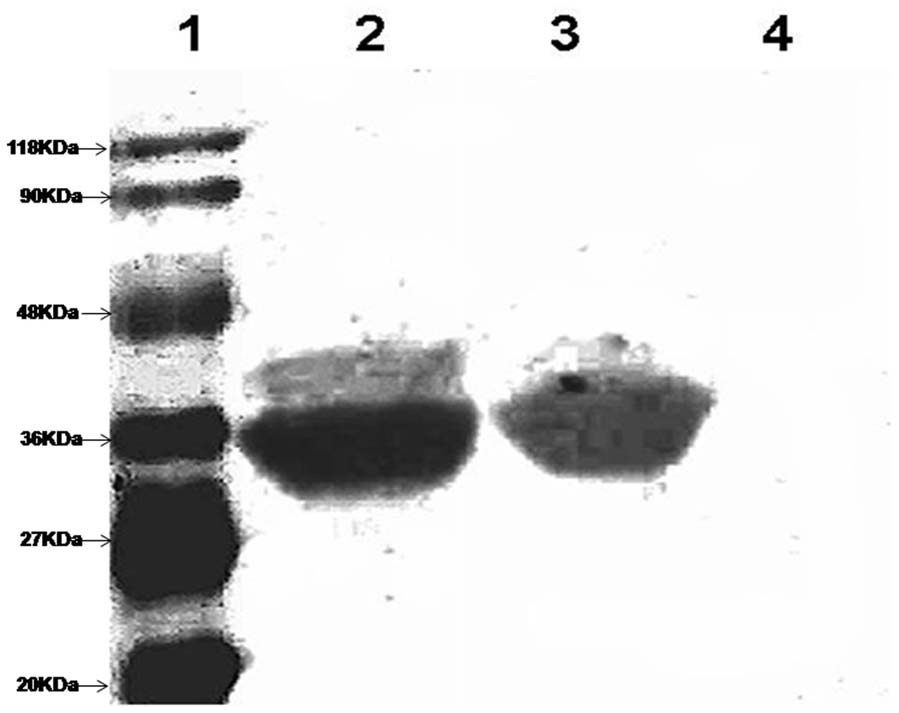
Biotinylation of GST-HTH domain by *Ec*BirA or *Mt*BPL. 5 µM fusion protein was incubated with 500 µM biotin, 3 mM ATP, 100 nM *Ec*BirA in standard buffer for 1 h. The sample was then resolved on 10% SDS-PAGE and transferred to nitrocellulose membrane and the biotinylated protein was detected by streptavidin HRP and H_2_O_2_. (1) marker ; (2) GST-HTH fusion protein (pGEN1)+100 nM *Ec*BirA; (3) GST-HTH fusion protein (pGEN1)+50 nM *Ec*BirA (4) GST – HTH fusion protein+100 nM *Mt*BPL.

*Ec*BirA has an additional N-terminus HTH domain which contributes to the repressor function of the protein (Figure 2). Earlier reports suggested that truncated *Ec*BirA (Δ1–34) was enzymatically active but did not undergo self-biotinylation [22]. This suggested that the N-terminus probably carries the biotinable residues. So, the N-terminus domain (1–65 amino acids) was independently cloned in pGEX4T-1. The fused GST-HTH domain of *Ec*BirA (pGEN1) was subjected to biotinylation using enzymatic concentration of 100 nM *Mt*BPL/*Ec*BirA. The fused protein was biotinylated by full length *Ec*BirA (Figure 4). The control GST protein was not biotinylated by *Ec*BirA. This confirms that the self-biotinable lysine is within the N-terminus/HTH domain of *Ec*BirA. It also suggests that the catalytic and self biotinable domain require no physical contiguity for the covalent modification. Hence, this construct was used to investigate if the lack of self biotinylation in *Mt*BPL was because (i) *Mt*BPL lacks the N-terminus domain or (ii) the enzyme was deficient in promoting self-biotinylation. *Mt*BPL failed to biotinylate HTH–GST fusion protein (pGEN1) but *Ec*BirA efficiently biotinylated the fusion protein. This suggests that the mere presence of self - biotinable residue does not confer *Mt*BPL an ability to self biotinylate. Furthermore, non-specific proteins such as BSA was biotinylated by *Ec*BirA but not by *Mt*BPL (Fig S2). This clearly reinstates that *Mt*BPL does not catalyze indiscriminate biotinylation. Thus, the inability of *Mt*BPL to undergo self-biotinylation could be attributed to two factors: *absence of an HTH domain* and *a stringent catalytic specificity of the enzyme*. (Figure 4).

### Competitive inhibition of self - biotinylation by Schatz peptide

**Figure 5:**
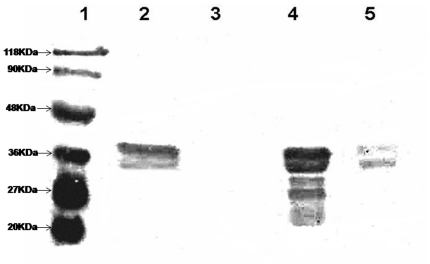
Competitive inhibition of GST-HTH protein and *Ec*BirA in the presence of excess amount of Schatz peptide. GST-HTH (5 µM) was incubated with biotin, ATP and 100 nM *Ec*BirA or *Ec*BirA (2 µM) was incubated with biotin, ATP in the presence/absence of Schatz peptide for 1 h at 37°C. The biotinylated proteins were detected by streptavidin HRP. (1) Protein marker; (2) GST-HTH protein; (3) GST-HTH+Schatz peptide; (4) *Ec*BirA; (5) *Ec*BirA+Schatz peptide.

The intermediate molecule, bio-5′AMP, appears to play a central role in several processes. We investigated if bio-5′AMP was preferentially used for self-biotinylation of HTH domain or biotinylation of biotin acceptor molecule. For this, self-biotinylation of *Ec*BirA was performed in the presence of saturated concentration of Schatz peptide or BCCP_87_ (5 µM). *Ec*BirA failed to undergo self-biotinylation or promote biotinylation of heterologous HTH (pGEN1) domain in the presence of excess biotin acceptor molecule such as Schatz peptide (Figure 5). Indeed, the bio-5′AMP synthesized was used for biotinylation of biotin acceptor molecules, Schatz peptide and BCCP, rather than for self-biotinylation. Also to confirm that the covalently modified self-biotinylated *Ec*BirA was dialyzed to remove unbound biotin and ATP and then incubated with Schatz peptide. The covalently modified self-biotinylated *Ec*BirA failed to endogenously biotinylate Schatz peptide. However, the addition of biotin and ATP to previously self-biotinylated *Ec*BirA led to the conversion of apo-Schatz peptide to biotinylated form (Fig S3).

### Mutation analysis

Choi-Rhee *et al* have shown that the affinity of R118G mutant of *Ec*BirA for biotin decreased by ∼100 fold and the self-biotinylation increased several fold [22]. However, for the homologous R69A mutant of *Mt*BPL the binding constant for biotin was nearly the same as that observed for the wild type protein (data not shown) . Also, the R69A mutant of *Mt*BPL did not undergo self-botinylation (Lane 12, Figure S1). This highlights the differences in the structural and functional organization of *Ec*BirA and *Mt*BPL.

### Limited proteolysis

**Figure 6:**
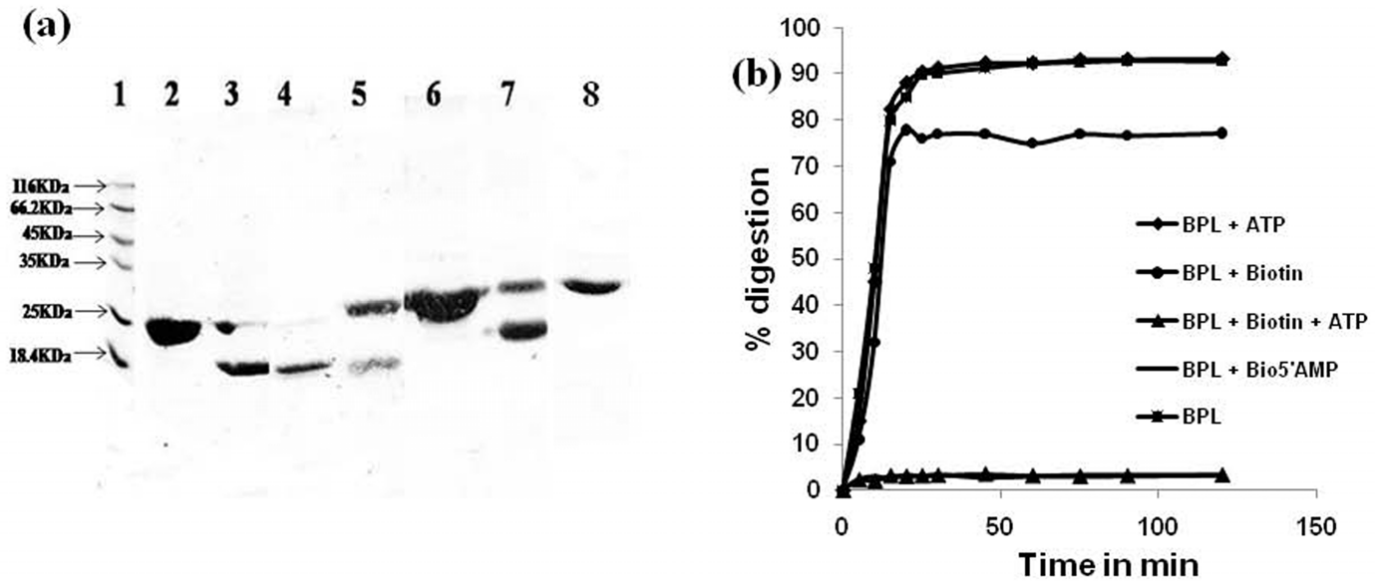
Limited proteolysis of *Mt*BPL by trypsin. *Mt*BPL (10 µM) was incubated with trypsin at 1;100 concentration for 30 min . The digested samples were resolved on a 10% SDS PAGE and the pattern observed by Coomassie blue stain.. The enzyme was pre-incubated with the substrates for 30 min prior to proteolysis by trypsin. Molecular weight marker (2) BPL, no trypsin (3) BPL (4) BPL+ATP (5) BPL+biotin (6) BPL+biotin+ATP (7) BPL+biotin+non-hydrolyzable AMP pNPP (8) BPL+bio-5′AMP. (b) The percentage of digestion during a period of 2 h. *Mt*BPL was pre-incubated with substrates 500 µM biotin, 3 mM ATP, biotin+ATP, 10 µM bio-5′AMP for 30 min and then subjected to proteolysis by incubating with trypsin for 20 min.

Purified *Mt*BPL was subjected to proteolytic digestion with protease trypsin for 20 min and the products were analyzed on 12% SDS PAGE in order to define the domain boundaries within the enzyme. The enzyme was subjected to limited proteolysis in the presence and absence of biotin and MgATP. Trypsin generated two fragments, one of about ∼8.2 kDa and the other of ∼21 kDa as determined by N-terminus sequencing and SDS-PAGE (Figure 6a, b). The ∼8.2 kDa has an N terminus His-tag which was identified by its reactivity with the anti-His antibody. Also, the ∼8.2 kDa fragment was susceptible to further proteolysis. The N-terminus sequencing of these products revealed the cleavage occurred between Arg-72 and Gly-73 for trypsin. Since these cleavage points are located around the conserved biotin binding site (GRGRHGR), *Mt*BPL was subjected to proteolytic digestion in the presence of saturating amounts of the substrates, biotin and ATP as well as both of them together. Incubation with ATP did not alter the cleavage by trypsin with 83% of the protein being digested. Incubation with biotin did reduce the proteolysis with nearly 40% of the protein intact. However, incubation of *Mt*BPL with both biotin and ATP completely protected nearly all the protein from proteolytic digestion by trypsin. This was also observed when the protein was pre-incubated with chemically synthesized bio-5′AMP. In fact the intermediate molecule, biotinyl-5′AMP protected the protein from proteolytic digestion for over 24 h. Thus, when biotin and ATP were pre-incubated with the enzyme, biotinyl-5′AMP was synthesized and this intermediate molecule protected the protein from proteolysis by binding to the active site of the enzyme. *Mt*BPL was incubated with saturating amounts of biotin and non-hydrolyzable ATP analogue AMPpNpp and then treated with trypsin. The protein showed reduced protection against the protease as the non-hydrolyzable ATP analog failed to synthesize biotinyl-5′AMP. Taken together, these results suggest that the binding of the substrates and/or the formation of the intermediate, biotinyl-5′AMP, protects BPL from protease cleavage.

## Discussion

Acetyl CoA carboxylase of *M. tuberculosis* belongs to the class of heteromeric *ACCases* which are multi-domain, multi-subunit enzyme. The subunit assembly of *acc*A3 and *acc*D6 complex in association with ε- subunits has been studied in detail [8]. The BCCP domain of heavier α (*accA*) subunit interacts with three distinct heterologous proteins; BCCP-BC, BCCP-CT and BCCP-BPL. Considering the complexity of the cell wall of *M. tuberculosis*, it is not surprising that the pathogen has so many of these enzymes with biotinyl domains.

BCCP is a key player in carboxylation and transcarboxylation reactions which shuttles carboxyl group from BC to CT of ACC to initiate fatty acid elongation. As a prelude to carboxylation of biotin to transcarboxylation of acyl-CoA, BPLs must selectively interact with BCCP. Relating structure to function of a protein that participates in multiple interactions is fraught with difficulties [23], [24]. From the crystal structures of *Ph*BCCP and *Ec*BCCP, it was evident that target lysine is located at the type 1 β- turn [11], [25]. In most post-translational modifications, the primary structure surrounding target residue is critical. But from the biotinylation results of this study it is apparent that while the motif is necessary it is not enough for biotinylation. Indeed failure of *Mt*BPL to biotinylate *Ec*BCCP_87_ is consistent with this argument. Hence, specific conformational feature(s) around the motif are necessary for biotinylation of the acceptor domain.

We reported earlier that BCCP domain of *accA1* was efficiently biotinylated and hence probably participates in the acetyl CoA carboxylase activity [20]. *M. tuberculosi*s has three BCCP domains each one belonging to a biotin carboxylase paralog. Our interest was to study the specificity of *Mt*BPL for the reactive biotinable lysine residue(s). This was of interest especially considering that *Ec*BirA could biotinlate BCCP from *S. cerevisiae*. Our study clearly defines the substrate specificity of *Mt*BPL. The gram positive protein ligase could not biotinylate Schatz peptide or *Ec*BCCP at all the conditions tested. In contrast, *Ec*BirA could biotinylate Schatz peptide and also *Mt*BCCP showing broad substrate specificity. Association of BirA –*BCC*P is complex and in *E. coli*, a cysteine residue in the conserved hydrophobic patch (LCIV) of β4-β5 turn promotes dimerization of apo-*Ec*BCCP. On biotinylation, the cysteine residue is buried contributing to monomerization of holo-*Ec*BCCP [12]. However, *Ph*BCCP and *Mt*BCCP lack this crucial cysteine residue. The C-terminus of BPL undergoes relatively large conformational changes to accommodate BCCP [13]. The BCCP domains from different species have varied structural organization to interact with their homolgous enzyme(s) [26], [27]. Display of a stringent specificity for its substrate is probably very critical for *Mt*BPL due to the presence of different paralogs of BCCPs (*accA1*, *accA2*, *accA3*) in its genome.

While Choi Rhee *et al* showed that R118G mutant of *Ec*BirA promotes self- biotinylation and also biotinylates BSA, we show that wild type *Ec*BirA itself at higher concentration of ATP and biotin exhibited self-biotinylation. It also promoted promiscuous biotinylation of BSA. In contrast, *Mt*BPL did not undergo self- biotinylation nor promote appreciable promiscuous biotinylation of BSA (Fig S1, S2, and S3).

Certain BPLs have a flexible active site domain that accommodates different substrates. Though *Ec*BirA and *Mt*BPL share considerable sequence homology they differ in their activities in a fundamental manner. A profound difference between *Ec*BirA and *Mt*BPL is self-biotinylation exhibited by the former enzyme. The N-terminus domain (HTH domain) of *Ec*BirA is the site of self- biotinylation. Δ1–34 *Ec*BirA failed to undergo self-biotinylation [22]. Also, biotinylation of heterologus pGEN1 (1–65 amino acid N-terminus domain of *Ec*BirA) confirmed that HTH domain had the self- biotinable lysine residue. As mentioned earlier, *Mt*BPL failed to undergo self-biotinylation probably because it lacks the HTH (repressor) domain. Sequence analysis showed that *Ec*BirA, *Ph*BPL and *Aa*BPL have **18**, **25** and **16** lysine residues compared to just **2** residues in *Mt*BPL. The two lysine residues of *Mt*BPL are within the conserved ‘KWPND’ and ‘KIAGLEV’motifs and are probably part of the active site. The invariant lysine within the KIAGLEV plays an essential catalytic role during synthesis of bio-5′AMP and the KWPND shares the motif with streptavidin. Thus, the lack of self-biotinylation in *Mt*BPL is due to the absence of a biotinable lysine residues. The specific lysine residues involved in the self-biotinylation of the HTH domain are currently under investigation in our laboratory. In biotinylation of BCCP, an electrostatic interaction between negative phosphate group of bio-5′AMP and positively charged lysine of BCCP are key elements. The uncharged lysine in BCCP is deprotonated by aspartate residues of *Ec*BirA which promotes a nucleophilic attack on the electrophilic carbonyl group of bio-5′AMP leading to covalent modification [27]. It is possible that a similar mechanism promotes self-biotinylation of HTH domain of *Ec*BirA. However, the self-biotinable residues in BPLs may not have sufficient accessibility and reactivity for accepting biotin and hence require longer incubation which perhaps accounts for a lag period of 1 h. *Intermolecular* interaction of BPL and BCCP probably allows for a snug fit which in turn promotes a fast and efficient covalent modification of the acceptor target lysine in BCCPs. On the other hand *intramolecular* folding of BPL initiated by bio-5′AMP may impart steric hindrance which probably restrains orientation of the adenylate towards the self-biotinable lysine.

Intramolecular folding in *Ec*BirA enables deprotonation of self-biotinable/promiscuous biotinable lysine residue leading to its covalent modification. However, the transition state of *Mt*BPL probably selects the specific acceptor molecule which in turn explains its stringent specificity for its cognate BCCP. Studies reported show that *Mt*BPL differs from *Ec*BirA and probably other BPLs in many additional ways; (a) *Mt*BPL is a monomer in both its apo and holo forms and has relatively lower affinity for biotin and bio-5′AMP (b) In *Ec*BirA, self-biotinylation was enhanced in R118G mutant which releases bio-5′AMP leading to increased self-biotinylation of the mutant protein. The R69A mutant of *Mt*BPL failed to undergo self- biotinylation suggesting that the proclivity of the enzyme for biotinylation was different from that of *Ec*BirA. The R69A *Mt*BPL has similar affinity for biotin as that of wild type in contrast to R118G *Ec*BirA which exhibited reduced affinity for biotin. Self-biotinylation of *Ec*BirA occurs only in the absence of a biotin acceptor molecule. This is of relevance to the repressor function of *Ec*BirA which occurs only in the absence of biotin acceptor molecule. Limited proteolysis study further reveals that the folding of the ligases are different. Our studies show that *Mt*BPL is cleaved at the N-terminus (72–73 amino acids) whereas *Ec*BirA is known to be cleaved at the C-terminus (217–218 amino acids) [28]. *Mt*BPL exhibit restricted cleavage in the presence of substrate suggesting that scissile site interacts with the substrate. While the biotin binding site is constituted by the conserved ‘GRGRHGR’ in both the BPLs but binding of biotin/bio-5′AMP promotes conformational change in *Ec*BirA [28], [29].

Self-biotinylation is intrinsic to the catalytic function of the given BPL as availability of the self biotinable domain of *Ec*BirA (pGEN1) does not promote promiscuous biotinylation by *Mt*BPL. In support, *Aa*BPL which lacks the HTH domain undergoes self-biotinylation at higher enzyme concentration (>500 nM) as observed with *Ec*BirA [30], [31]. In *Mt*BPL, the lack of self-biotinylation is due to both substrate stringency of the enzyme and also due to the lack of a target lysine residue. The absence of self-biotinylation in *Mt*BPL is probably a desirable feature to facilitate the high demands of fatty acid biosynthesis in *M. tuberculosis*. However, in other biotin protein ligases with or without the HTH domain, self-biotinylation is seen to take place.

### Proposed rationale for the diverse functional organization of BPLs

#### *Mt*BPL

**Figure 7:**
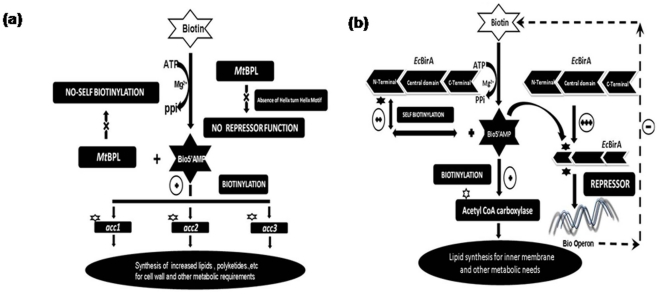
A schematic illustration proposing the mechanism of biotin utilization and their physiological significance. (a) Intrinsic metabolic functions of *Mt*BPL (b) Intrinsic metabolic functions of *Ec*BirA and their physiologic significance.

We reported earlier that *Mt*BPL in spite of lower affinity for biotin had *K_m_* similar to that of *Ec*BirA [20]. Deletion of N-terminus domain of *Ec*BirA decreases binding affinity of the enzyme by ∼100 fold [29]. This suggests that higher binding constant of *Ec*BirA for biotin may be directed towards covalent modification of HTH domain. In *Mt*BPL, fatty acid synthesis plays central role for its cell wall synthesis. As this is a rate limiting step, the enzyme avoids self/promiscuous biotinylation to conserve biotin, a scarce co-factor whose biosynthesis itself is an extremely slow process. This is due to the low turn over of *BioB* and its degradation under low iron concentration [32], [33]. Additionally, uncoupling biotinylation and repressor functions would favor fatty acid biosynthesis [34]. Hence, the mycobacterium cell probably reserves all the biotin at its disposal for biotinylation of *acc* to meet the demands of cell wall biosynthesis (Figure 7a).

#### *E. coli*

The intermediate molecule, bio-5′AMP can be utilized for any of the three function: biotinylation of BCCP, self-biotinylation or as a co-repressor depending on the cellular demands (Figure 7b).

1. At high BCCP concentration, low bio-5′AMP [+] mediates biotinylation of biotin acceptor molecule.
2. At low BCCP concentration and moderate bio-5′AMP [++] , when the cell does not require biotin for biotinylation reaction, bio-5′AMP [++] probably needs to functions as a co-repressor of biotin biosynthetic pathway and repress synthesis of biotin. However, this would be favored only if *E.coli* does not require immediate fatty acid biosynthesis to operate. But the bacterium during the transition, probably requires additional time to decide whether it wants to block the biotin biosynthetic pathway. Under such a situation, in the absence of BCCP, the bio-5′AMP is directed towards self-biotinylation. This prevents the bio-5′AMP to be utilized as a co-repressor of biotin biosynthetic pathway. The self- biotinylated *EcBirA* is enzymatically active to participate in the biotinylation of BCCP. This is primarily because transcription activation or repression has to be modulated according to the cellular requirements [34]
3. However, when the concentration of bio-5′AMP [+++] is abundant it functions as a co-repressor and shuts the biotin biosynthetic pathway.

Our results support the proposed hypothesis as self- biotinylation is competitively inhibited by biotin acceptor molecule which is is increased in the presence of operator sequence of biotin biosynthetic pathway [18].

The preferred order of bio-5′AMP utilization by *Ec*BirA is:
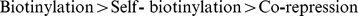
Thus the evolutionary process has devised different mechanism in *Ec*BirA and *Mt*BPL commensurate with the functional requirement of the organism. The biotin repressor function is separated from enzyme function in *Mt*BPL as lipid biosynthesis is very critical in *M. tuberculosis*. As the repressor function is not coupled to the enzyme function the enzyme does not promote self-biotinylation. However, in *E. coli* during the evolutionary process, the enzyme has probably compromised its substrate specificity and has also acquired self as well as promiscuous biotinylation.

Yao *et al* [35], [36] suggested that though functionality and overall folding of biotinyl domains are conserved through evolution, the detailed structures of BPL-BCCP binding interface may vary among different species. The substrate stringency of *Mt*BPL may add to its ability to regulate the acyl CoA carboxylases in *M. tuberculosis*.

In conclusion, our studies with *Mt*BPL show that biotinylation process is not dependent merely on recognition of a target residue but involves an intricate play between the biotinyl acceptor (BCCP) and its cognate ligase. *Mt*BPL plays an active role in substrate selection which occurs by an integration of an intricate series of events involved in BPL-BCCP interaction and biotin demands of the cell. The stringency exhibited by *Mt*BPL makes it a suitable target for the development of anti-mycobacterials and vaccine.

## Materials and Methods

### Protein methods

*M. tuberculosis* BPL ( Rv3279c) was cloned into pET28a at NdeI/HindIII sites and the protein purified as described by Purushothaman *et al* [20]. Mutant R69A was generated by site- directed mutagenesis and cloned into NdeI/HindIII sites and sequence analyzed. The procedure used for the purification of the mutant protein was identical to that of its wild type counter-part wild type [20]. *Ec*BirA and (Δ1–65) *Ec*BirA. *M. tuberculosis* has three acetyl-/-propionyl coenzymeA carboxylase α subunit *accA1* (*Rv2501c*), *accA2* (*Rv0973c*), *accA3* (*Rv3285c*), and a putative acetyl CoA carboxylase subunit BCCP *TB7.3* (*Rv3221c*) and six β subunit, *accD* genes [7], [8]. All biotinyl domains so far reported have target lysine at −35^th^ residue from C-terminus for biotinylation. Hence, we cloned the C-terminus 87 amino acid residues of *accA1* as the substrate for *Mt*BPL. *Mt*BCCP_87_and *Ec*BCCP_87_ were cloned into pET28a. The PCR primers used for amplification reaction are listed in Table 1.

**Table 1:** List of primers used.

Name	Sequence
*Mt*BCCP_87_ fwd	5′- GGAATTCCATATGCACCTGCGCGAGGCCGAGGA-3′
*Mt*BCCP_87_ rev	5′- CCCAAGCTTCTAGTCCTTGATCCTCGCCAGTACC-3′
*Ec*BCCP_87_fwd	5′-GGAATCCATGATGGAAGCGCCAGCAGCAGCGGAAATC-3′
*Ec*BCCP_87_rev	5′-CGCCTCGAGCTCGATGACGACCAGCGGCTCGTCAAATTC-3′
*Ec*BirA fwd	5′- GGAATTCCATATGATGAAGGATAACACCGTGCCACTGAAA-3′
*Ec*BirA rev	5′- CCAAGCTTTTATTTTTCTGCACTACGCAGGGATATTTCACC-3′
Tr*Ec*BirA fwd	5′ -GGAATTCCATATGCAGTTACTTAATGCTAAACAG-3′
Tr*Ec*BirA rev	5′- CCCAAGCTTTTATTTTTCTGCACTACGC -3′
R69A *Mt*BPL fwd	5′ – ATCGCCGAGCATCAGACCGCTGGGCGGGGGCCCATGGC -3′
R69A *Mt*BPL rev	5′- TCGGGCAGTGGCCGCCCAGCCGCGGCCATGGGCCCCCCG -3′

For self-biotinylation studies, BL21 expressing *Ec*BirA was grown in M9 minimal media supplemented with 2% glucose for 4 h and then induced with 100 µM IPTG for 3 h. This was carried out to prevent autologous self-biotinylation.

### Schatz minimal peptide

A minimal peptide, Schatz peptide, which is efficiently biotinylated by *Ec*BirA GLNDIFEAQKIEWH (Genscript, USA) [37], was used for some of the experiments (37). The peptide (5 µM) was incubated with 100 nM of *Ec*BirA/*Mt*BPL, biotin (500 µM), ATP (3 mM) for 1 h at 37°C in standard buffer and the biotinylation was detected by MALDI-TOF.

### Matrix-assisted laser desorption time of flight mass spectrometry

The molecular weight of Schatz and holo-Schatz peptides were determined by MALDI-TOF MS using a Ultraflex TOF/TOF , (Bruker Daltonics Germany) equipped with a N2 Laser, 337 nm, 50 Hz operating in the 25 KvA reflector mode. Samples were dialyzed against water and l µl of sample was mixed with equal volume of matrix solution on a stainless steel plate and air-dried prior to analysis. The matrix solution used was α-cyano-4-hydroxycinnamic acid in 50% acetonitrile, 0.1% (v/v) trifluoroacetic acid. Mixture of appropriate standards was used for calibration and Schatz and holo-Schatz peptide analytes were analyzed as described above and calibration was performed using the known protonated molecular ion (MH1).

### Fast Protein Liquid Chromatography

A reaction mixture of *Mt*BPL (20 µM), biotin (500 µM), ATP (3 mM) , MgCl_2_ (2.5 mM), and *Mt*BCCP_156_ (20 µM) were incubated for 30 min at 37°C and then 200 µl of the reaction mixture was loaded onto Superdex S200 (GE, Healthcare) and eluted at a flow rate of 0.2 ml/min and the eluted samples were monitored at 280 nm. The gel filtration column was calibrated with alcohol dehydrogenase, BSA, ovalbumin, carbonic anhydrase and chymotrypsin and lysozyme. Also, purified *Mt*BCCP_156_ was loaded on to the column to determine the oligomeric status of apo-*Mt*BCCP_156_.

### Biotinylation assay

Biotin acceptor molecule (BCCP or Schatz peptide) were incubated with 500 µM biotin, 3 mM ATP, 2.5 mM MgCl_2_ and 100 nM *Mt*BPL or *Ec*BirA in standard buffer for 1 h at 37°C. The biotinylated proteins were detected by avidin blot and mass spectrometry..

### Self-biotinylation reaction

To determine self-biotinylation, different concentration of *Ec*BirA/*Mt*BPL were incubated with 3 mM (ATP), biotin (500 µM) in standard buffer for 1 h at 37°C. The biotinylated protein was then detected by streptavidin blot.

### Avidin blot

Biotinylated proteins were resolved on 10% SDS-PAGE and transferred to nitrocellulose membranes. The non-specific sites were blocked with 5% skim milk in Phosphate buffered saline +0.1% Tween20, pH-7.4, (PBS-T) and incubated with streptavidin-HRP (Sigma) at 1∶2000 dilution for 1 h at room temperature. The membrane was washed 5× with PBS-T and 2× with PBS and detected by 3-amino-9- ethylcarbazole (AEC)/H_2_O_2_.

### Limited proteolysis

*Mt*BPL (5 µM) was incubated with trypsin (1∶200) dilution and incubated at 37°C for 20 min. The enzyme was pre-incubated with biotin (500 µM) and ATP (3 mM) at 37°C for 30 min prior to trypsin digestion. After protease treatment, the sample solubilizing dye was added to the protein , boiled and loaded on to a 10% SDS-PAGE. The resolved proteins were scanned and percentage of proteolysis determined. The digested product was sequenced from the N-terminus on an Applied Biosystems Precise 491 CLC Protein Sequencer.

## Supporting Information

Figure S1 Self-biotinylation of *Ec*BirA, *Mt*BPL and R69A *Mt*BPL mutant by avidin blot. *Mt*BPL/*Ec*BirA (250–2000 nM)/R69A (2000 nM) were incubated with 3 mM ATP and 500 µM biotin in standard buffer (10 mM Tris- HCl pH-8.0, 50 mM KCl, 2,5 mM MgCl_2_ ) for 1 h at 37°C. The reaction mixture was resolved on a 10% SDS PAGE and transferred to nitrocellulose membrane. The membrane was then incubated with streptavidin HRP for 1 h at room temperature and developed with AEC/H_2_O_2. (1)_ marker; (2–6) 250–2000 nM of *Mt*BPL; (7–11) 250–2000 nM of *EcBirA*; (12) 2000 nM of R69A *Mt*BPL. See also Figure S2.
(TIF)

Figure S2 Promiscuous biotinylation property of *Ec*BirA and *Mt*BPL by avidin blot. BSA (2 µM) were incubated with 3 mM ATP, 500 µM biotin and 100 nM BPL in standard buffer (10 mM Tris-HCl pH-8.0, 50 mM KCl, 2.5 mM MgCl_2_) for 2 h at 37°C. The reaction mixture was then resolved on a 10% SDS PAGE and transferred to nitrocellulose membrane. The membrane was then incubated with streptavidin HRP for 1 h at room temperature and developed with AEC/H_2_O2. (1) marker (2) BSA+400 nM *Mt*BPL; (3–5) BSA+200, 300, 400 nM of *Ec*BirA.
(TIF)

Figure S3 Catalytic activity of self-biotinylated *Ec*BirA . Self-biotinylated *Ec*BirA was dialyzed to remove free biotin/ATP. The enzyme was then used to transfer biotin to Schatz peptide in the absence or presence of endogenous biotin and ATP. (a) Mass spectrum of Schatz peptide incubated with self-biotinylated *Ec*BirA in standard buffer. (b) Mass spectrum of Schatz peptide incubated with self-bitoinylated *Ec*BirA incubated with endogenous 500 µM biotin, 3 mM ATP and in standard buffer.
(TIF)
