# Supplementary figures and images for: Diversity in Functional Organization of Class I and Class II Biotin Protein Ligase
Source: PLoS One. 2011 Mar 3;6(3):e16850. doi: 10.1371/journal.pone.0016850 (PMC3048393; doi:10.1371/journal.pone.0016850)

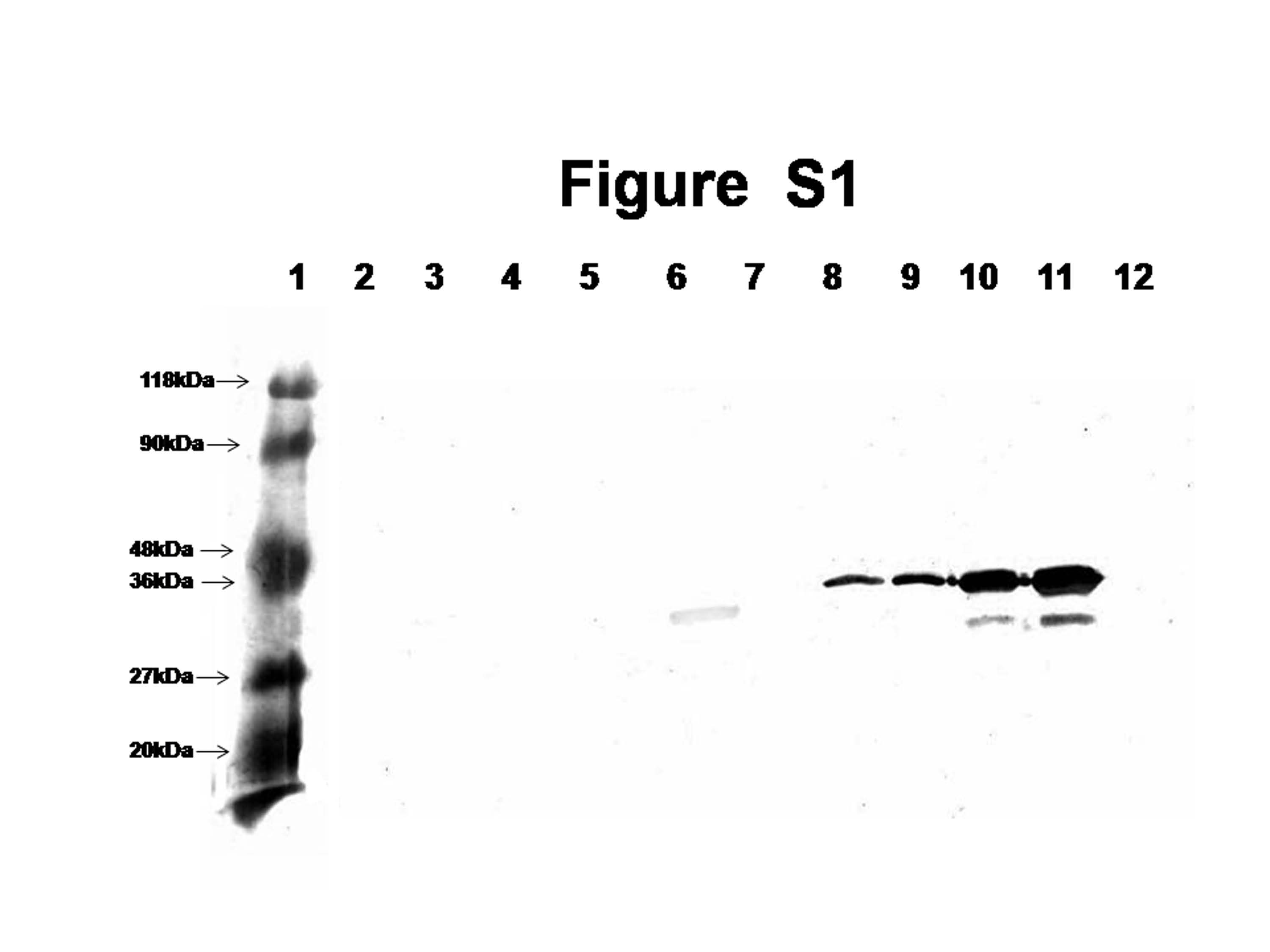

Supplement: Figure S1 — Self-biotinylation of EcBirA, MtBPL and R69A MtBPL mutant by avidin blot. MtBPL/EcBirA (250–2000 nM)/R69A (2000 nM) were incubated with 3 mM ATP and 500 µM biotin in standard buffer (10 mM Tris- HCl pH-8.0, 50 mM KCl, 2,5 mM MgCl2 ) for 1 h at 37°C. The reaction mixture was resolved on a 10% SDS PAGE and transferred to nitrocellulose membrane. The membrane was then incubated with streptavidin HRP for 1 h at room temperature and developed with AEC/H2O2. (1) marker; (2–6) 250–2000 nM of MtBPL; (7–11) 250–2000 nM of EcBirA; (12) 2000 nM of R69A MtBPL. See also Figure S2. (TIF) [file pone.0016850.s001.tif]

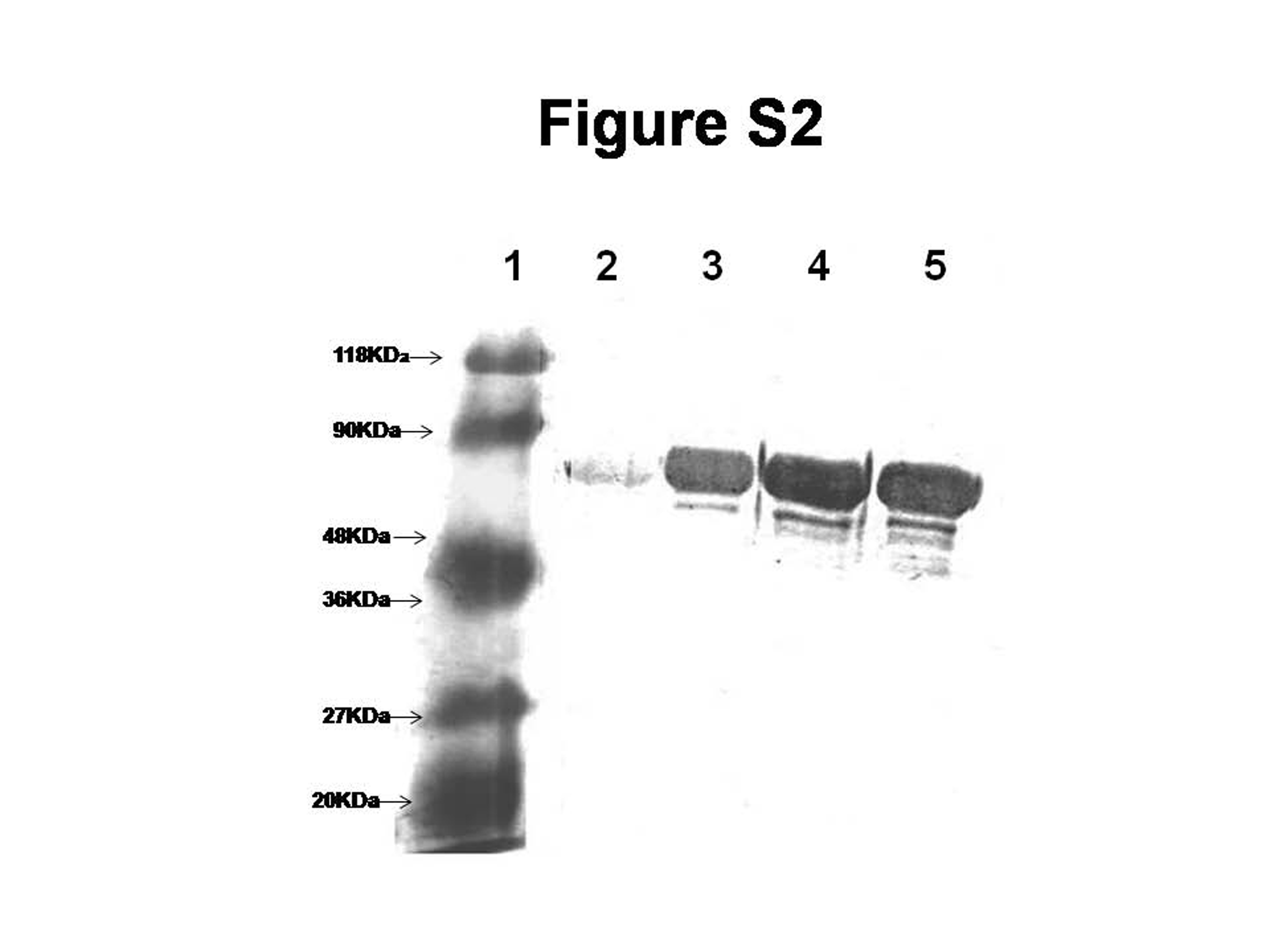

Supplement: Figure S2 — Promiscuous biotinylation property of EcBirA and MtBPL by avidin blot. BSA (2 µM) were incubated with 3 mM ATP, 500 µM biotin and 100 nM BPL in standard buffer (10 mM Tris-HCl pH-8.0, 50 mM KCl, 2.5 mM MgCl2) for 2 h at 37°C. The reaction mixture was then resolved on a 10% SDS PAGE and transferred to nitrocellulose membrane. The membrane was then incubated with streptavidin HRP for 1 h at room temperature and developed with AEC/H2O2. (1) marker (2) BSA+400 nM MtBPL; (3–5) BSA+200, 300, 400 nM of EcBirA. (TIF) [file pone.0016850.s002.tif]

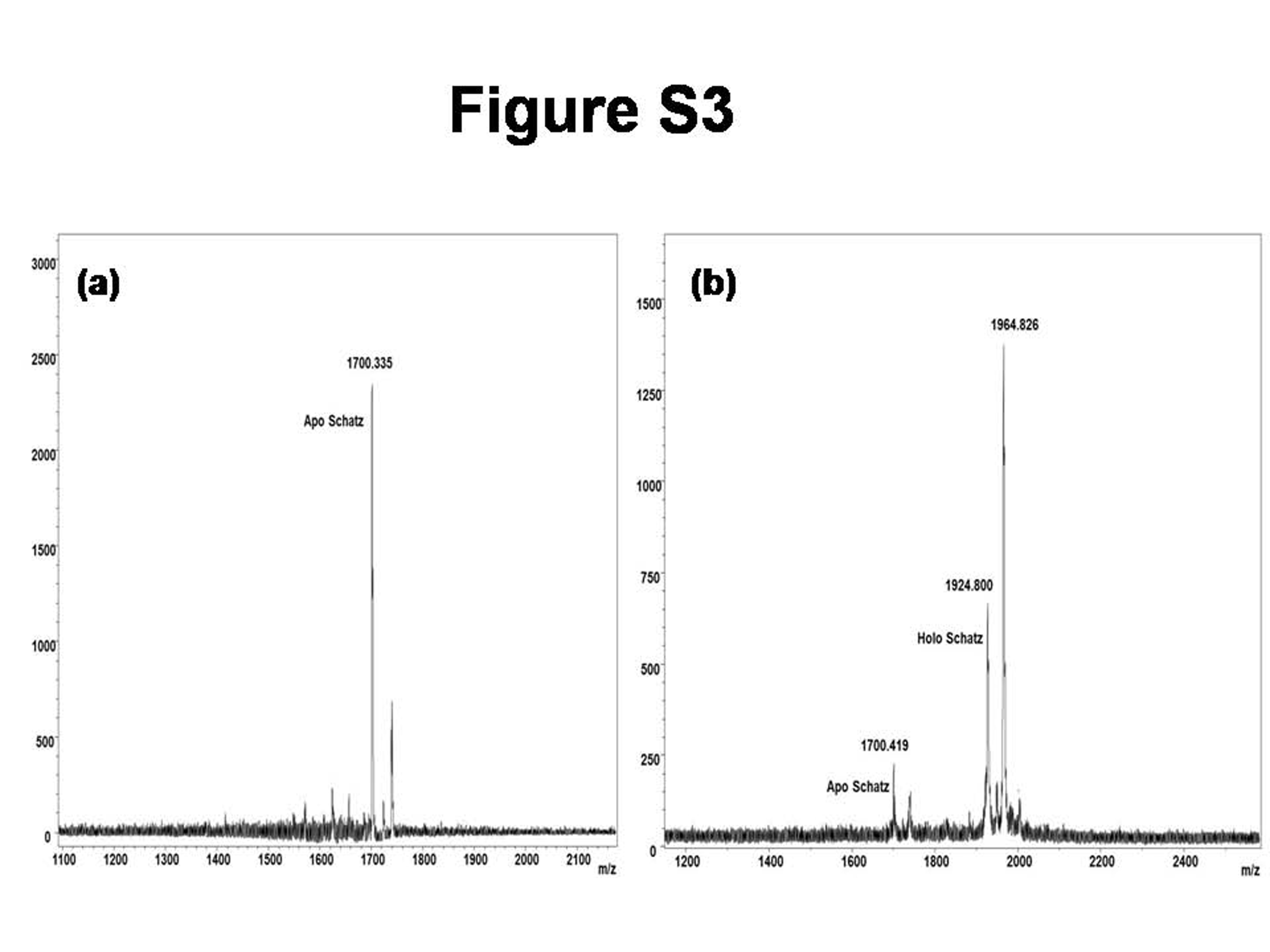

Supplement: Figure S3 — Catalytic activity of self-biotinylated EcBirA . Self-biotinylated EcBirA was dialyzed to remove free biotin/ATP. The enzyme was then used to transfer biotin to Schatz peptide in the absence or presence of endogenous biotin and ATP. (a) Mass spectrum of Schatz peptide incubated with self-biotinylated EcBirA in standard buffer. (b) Mass spectrum of Schatz peptide incubated with self-bitoinylated EcBirA incubated with endogenous 500 µM biotin, 3 mM ATP and in standard buffer. (TIF) [file pone.0016850.s003.tif]
